# Severe Acute Respiratory Syndrome Coronavirus 2 Variant Infection Dynamics and Pathogenesis in Transgenic K18-h*ACE2* and Inbred Immunocompetent C57BL/6J Mice

**DOI:** 10.3390/v17040500

**Published:** 2025-03-30

**Authors:** Hongwei Liu, Brianna M. Ramirez, Talia S. Wong, Christopher M. Weiss, Kevin C. K. Lloyd, Qizhi Gong, Lark L. Coffey

**Affiliations:** 1 Department of Pathology, Microbiology, and Immunology, School of Veterinary Medicine, University of California, Davis, CA 95616, USA; 2 Department of Cell Biology and Human Anatomy, School of Medicine, University of California, Davis, CA 95616, USA; brirami@ucdavis.edu (B.M.R.); qzgong@ucdavis.edu (Q.G.); 3 Mouse Biology Program, University of California, Davis, CA 95616, USA; 4 Department of Surgery, School of Medicine, University of California, Davis, CA 95616, USA

**Keywords:** COVID-19, SARS-CoV-2, infection dynamics, pathogenesis, mouse model, transgenic mice, human ACE2 receptor-expressing mice, K18 mice, C57BL/6J mice

## Abstract

The global impact of the COVID-19 pandemic, caused by severe acute respiratory syndrome coronavirus 2 (SARS-CoV-2), persists in part due to the emergence of new variants. Understanding variant-specific infection dynamics and pathogenesis in murine models is crucial for identifying phenotypic changes and guiding the development of countermeasures. To address the limitations of earlier studies that investigated only a few variants or used small sample sizes, we evaluated clinical disease, infection kinetics, viral titers, cellular localization, and histopathologic changes in the lungs and brains of transgenic B6.Cg-Tg(K18-*ACE2*)2Prlmn/J (“K18”) and corresponding genetic control (C57BL/6J) mice expressing human angiotensin-converting enzyme 2 (hACE2). Six SARS-CoV-2 variants were assessed: B.1 (WA1-like), alpha, beta, delta, omicron, and omicron XBB.1.5, using cohorts of ≥18 mice. Following intranasal inoculation with B.1, alpha, beta, or delta variants, K18 mice experienced rapid weight loss and reached euthanasia criteria by 5–6 days post-inoculation (dpi). In contrast, K18 mice inoculated with both omicron variants recovered to their starting weight within 4–6 dpi. Infectious SARS-CoV-2 was detected in the oropharynx at 1 and2 dpi, in the lungs at 2, 4, and 6 dpi, and in the brain at 4 and 6 dpi for all variants except omicron. SARS-CoV-2 nucleoprotein was detected, and interstitial pneumonia of varying severity was observed in K18 mice infected with all variants. Brain lesions were identified in mice infected with the B.1, beta, and delta variants 6 dpi. As K18 mice express hACE2 in the brain—a feature not present in humans—we also compared infection dynamics of three variants to those of a mouse-adapted WA1 strain in C57BL/6J mice lacking the human *ACE2* gene. C57BL/6J mice did not experience lethal disease, exhibited milder pneumonia, and had no evidence of neuroinvasion despite similar infection kinetics to K18 mice. These findings demonstrate contrasting phenotypes across the two models and reduced tropism and pathology of omicron compared to earlier variants in both models. This comprehensive analysis of SARS-CoV-2 variants in two mouse models provides valuable insights for model and variant selection for future studies.

## 1. Introduction

The global emergence of severe acute respiratory syndrome coronavirus 2 (SARS-CoV-2) in 2020 led to the COVID-19 pandemic that caused nearly 800 million cases as of January 2025 [1,2]. COVID-19 leads to hospitalization rates of 3–20% among diagnosed cases [3]. SARS-CoV-2 infection affects several organ systems including the lungs and causes symptoms including cough, fever, and headache. Severe cases can progress to COVID-19-associated acute respiratory distress syndrome, which can be fatal. Individuals with severe disease develop diffuse alveolar damage that disrupts oxygen exchange and causes shortness of breath, often requiring ventilatory support [3]. Post-COVID-19 condition, commonly known as long COVID, occurs in people who experience symptoms 3 months after initial SARS-CoV-2 infection that last at least 2 months [4]. Long COVID impacts up to 20% of SARS-CoV-2-infected people.

SARS-CoV-2 is a positive-sense single-stranded RNA virus in the Betacoronavirus family. Although the World Health Organization (WHO) declared an end to the COVID-19 pandemic in May 2023, the virus continues to evolve and circulate globally. Despite widespread vaccination and therapeutic efforts, new variants have emerged with different levels of infectivity, pathogenicity, and immune escape, which may undermine vaccine effectiveness and cross-protection [5,6,7,8]. Since the initial pandemic caused by the WA1-like (B.1) variant, detected in early 2020, the WHO has recognized several variants of concern (VOCs) that have dominated at different times, including alpha (B.1.1.7, first detected in September 2020), beta (B.1.351, May 2020), delta (B.1.617.2, October 2020), omicron (B.1.529, November 2021), and omicron-XBB.1.5 (late 2022) [9]. For each of these variants, it is critical to understand their infectivity, tissue tropism, and pathogenesis in order to assess the full spectrum of disease severity they produce and to define changes in disease phenotype and severity caused by different variants.

Animal models including ferrets, hamsters, non-human primates, and mice have been essential for COVID-19 research, providing insights that cannot be obtained from human or cell studies alone. Each animal model balances advantages and limitations including infectivity, transmission competence, tissue tropism, immune involvement, throughput, and cost [10,11,12,13,14]. Mice, in particular, are a desirable infectious disease model due to their small size, ease of breeding and genetic manipulation, and the availability of immunological tools. However, C57BL/6J mice, a common and well-characterized inbred immunocompetent mouse strain, are not susceptible to early SARS-CoV-2 variants due to an incompatibility in virus binding to mouse angiotensin-converting enzyme 2 (ACE2), a receptor necessary for viral entry [10,15]. This led to the widespread adoption of transgenic or humanized mice that express human ACE2 [5,13,16,17,18,19,20,21,22,23], including a model previously developed for SARS-CoV [15], which are susceptible to SARS-CoV-2 infection including in the upper and lower airway tissues that are hallmarks of human infection [10]. Among these, the keratin-18 humanized ACE2 (K18-h*ACE2*, hereafter referred to as “K18”) mouse model became prominent for its ability to replicate severe disease and was used for development of currently approved vaccines [24]. However, rapid mortality in K18 mice limits their use in long-term studies, which are crucial given the emergence of long COVID [10,11,16,17,25]. Additionally, SARS-CoV-2 infection in K18 mice leads to viral replication in the brain and the development of neurological sequelae and uniform mortality, which is not observed in humans [10,13]. However, COVID-19 is now recognized as a systemic illness with numerous neurologic manifestations and lingering morbidity resulting from indirect impacts to nervous tissues and COVID-19-mediated vasculopathies (reviewed in [26]). Despite these differences between humans and mice, the K18 model continues to provide valuable insights into severe disease progression, lung damage, and vaccine efficacy [5,18,19].

Due to the insusceptibility of C57BL/6J mice to early SARS-CoV-2 variants, mouse-adapted (MA) viruses were initially generated by serial passage in C57BL/6J mice to enable infection [10,27,28,29]. Subsequent studies demonstrated that beta and omicron infect C57BL/6J mice [27,30,31,32,33], allowing for the study of VOC infection without the need for genetic modifications to the host or the virus.

VOCs produce different phenotypes and severity in both K18 and C57BL/6J mice. For example, alpha and delta have demonstrated increased pathogenicity compared to B.1 in K18 mice [19]. Some VOCs encode mutations in the SARS-CoV-2 spike protein that enhance virus binding affinity to human and mouse ACE2, such as N501Y [5,31,34]. Unlike K18 mice, C57BL/6J mice infected with MA SARS-CoV-2 or permissive VOCs exhibit mild to moderate disease [27], similar to the spectrum of disease severity observed in humans. While previous studies have investigated individual VOCs [5,18,19,20,32], none have comprehensively compared multiple VOCs, including omicron-XBB.1.5, in K18 and C57BL/6J mice under the same experimental conditions. Variations in protocols, VOC strains, group sizes, mouse ages, virus doses, and sampling times across laboratories limit direct comparisons between studies. To address this gap, we evaluated acute infection dynamics of six SARS-CoV-2 variants—ancestral WA1-like (B.1), alpha (B.1.1.7), beta (B.1.351), delta (B.1.617.2), and two omicron strains (B.1.529 and XBB.1.5)in K18, and three variants, alpha, beta, and omicron XBB.1.5 plus MA SARS-CoV-2, in C57BL/6J mice through a retrospective analysis of control animals used in efficacy studies for candidate COVID-19 therapeutics infected via the same dose and route, and handled and longitudinally sampled in the same way across all experiments. Using a targeted combination of virology and histopathology, we provide a comprehensive comparison of SARS-CoV-2 VOC disease phenotypes in the most commonly used inbred mouse models for evaluating vaccines and therapeutic interventions. Our findings reveal variable infectivity of SARS-CoV-2 variants in K18 mice, with all VOCs except omicron causing lethal disease and high virus titers in the lung and brain. K18 mice showed more severe histopathologic changes in the lung and brain compared to C57BL/6J mice. This study underscores the importance of comparing SARS-CoV-2 variant phenotypes in murine models to better understand tropism and pathogenesis, which is crucial for selecting variants in future studies, including those evaluating therapeutic evaluations.

## 2. Materials and Methods

### 2.1. Ethics and Biosafety

All mouse work was conducted based on protocol #23489, approved by the institutional animal care and use committee (IACUC) at the University of California, Davis. Infectious virus was handled in certified animal biosafety level 3 laboratory (ABSL-3) spaces in compliance with approved institutional biological use authorization #R2813. The University of California, Davis, is accredited by the Association for Assessment and Accreditation of Laboratory Animal Care (AAALAC). All mouse work adhered to the NIH Guide for the Care and Use of Laboratory Animals.

### 2.2. Mice

The mice in this study were control animals used in efficacy studies for candidate COVID-19 therapeutics, which resulted in variable group sizes. Ordinary two-way ANOVA calculations on unequal group sizes were computed using the type III sum-of-squares method by default in GraphPad Prism version 10.4.1 to reduce bias. The minimum group size of 18 mice provides statistical power (0.8, α 0.05) to detect a mean difference in lung titer between 2 variants of 0.2 log_10_ PFU/tissue with a standard deviation of 0.2 log. Mice were used with an approximate equal sex ratio. Male and female 6–11-week-old B6.Cg.Tg(K18-*ACE2*) 2Prlmn/J (stock #34860) transgenic mice, in which a K18 transgene promotor (K18) was used to drive expression of the coding sequence of the human *ACE2* gene in a C57BL/6J congenic background, and C57BL/6J (stock #000664) mice aged 7–11 weeks old were purchased from Jackson Laboratories (Sacramento, CA, USA). All mice appeared healthy and had not been previously used for any other experiments. Mice of the same genetic background were co-housed by sex in ABSL-3 conditions in a ventilated isolation unit (Techniplast, West Chester, PA, USA) at 22–25 °C and a 12:12 h light/dark cycle with a maximum of 4 animals per cage and acclimated for up to 6 days. Rodent chow with 18% protein content (Envigo, Madison, WI, USA) and sterile bottled water were provided ad libitum for the duration of experiments.

### 2.3. Cell Lines and SARS-CoV-2 Isolates

African Green Monkey Kidney Epithelial Cells (Vero-E6, #NR-53728) expressing high endogenous angiotensin-converting enzyme 2 and Vero E6 expressing *ACE2* and *TMPRSS2* (Vero-E6-*TMPRSS2*-T2A-*ACE2*, #NR-54970) and Vero (CCL-81) were obtained from ATCC (Manassas, VA, USA) and BEI Resources, NIAID, NIH (Manassas, VA, USA), respectively. Vero-E6 and Vero CCL-81 cells were cultured at 37 °C with 5% CO_2_ in Dulbecco’s modified Eagle medium (DMEM; Gibco, Thermo Fisher Scientific, Emeryville, CA, USA) supplemented with 5% fetal bovine serum (FBS; Genesee Scientific, San Diego, CA) and 1× antibiotic–antimycotic (Fisher Scientific, Waltham, MA, USA). Vero-E6-TMPRSS2-T2A-ACE2 cells were cultured under the same conditions as the other Vero cell lines with the addition of 10 μg per mL puromycin (Fisher Scientific, Waltham, MA, USA). SARS-CoV-2 virus lineage B.1 (human/USA/CA-CZB-59 × 002/2020, GenBank #MT394528) isolated from a patient in 2020 in Northern California was generously provided by Dr. Christopher Miller; B.1.1.7 (hu/USA/CA_CDC_5574/2020, GISAID #EPI_ISL_751801), B.1.351 (hCoV-19/USA/MD-HP01542/2021, GISAID #EPI_ISL_89-360), B.1.617.2 (hCoV-19/USA/PHC658/2021, no GISAID number provided), B.1.1.529 (hCoV-19/USA/HI-CDC-4359259-001/2021, GISAID: EPI_ISL_8690072), omicron-XBB.1.5 (hCoV-19/USA/MD-HP40900/2022, GISAID: EPI_ISL_16026423), and B.1 MA-10 (USA-WA1/2020 backbone, GenBank: MT952602) were obtained from BEI Resources, NIAID, NIH (Table 1). All virus strains were propagated once after procurement in Vero CCL-81 cells to achieve titers of >10^6^ Vero plaque forming units (PFU)/mL, respectively. Single-use virus aliquots were stored at −80 °C until they were used in murine experiments.

### 2.4. SARS-CoV-2 Mouse Inoculation and Monitoring

Roughly equal numbers of male and female mice were randomly assigned to each treatment using a computerized random number generator. Mice were weighed and anesthetized with isoflurane, then inoculated intranasally (i.n.) via hanging drop over both nares with 30 μL Dulbecco’s phosphate-buffered saline (DPBS) or 1 × 10^4^ or 1 × 10^5^ PFU (total dose in both nares) of SARS-CoV-2 diluted in DPBS. Inocula were back-titrated by Vero plaque assay to confirm the target dose. Mice were monitored for changes in weight and clinical signs of disease including ruffled fur, ataxia, and labored breathing twice daily for up to 6 days post inoculation (dpi). Rodent handling, weighing, and sampling were standardized with a single unblinded operator to reduce variability. Each mouse was identified by ear punch and the sampling order of cages was randomized at the start of individual studies. Within each cage, mice were selected at random each day for sampling. The animal handler was not blinded to treatment groups. On 1 and 2 dpi, mice were anesthetized with isoflurane, and oropharyngeal samples were obtained by swabbing with rayon-tipped swabs (Puritan, Fisher Scientific, Waltham, MA, USA). Swabs were stored in 500 μL of DMEM containing 1% FBS and frozen at −80 °C. Swabs were centrifuged and liquid aspirated from the tube was used for titration. Mice were euthanized either at prescribed time points on 2, 4, and 6 dpi, when weight loss reached 20% of day-0 weight, or if animals were deemed moribund as evidenced by ataxia or rapid or depressed respiration rate. Prior to euthanasia, blood was collected by submandibular vein puncture under isoflurane anesthesia. Whole blood was clotted for >10 min at room temperature then centrifuged for 5 min at 8000× *g,* and separated serum was stored at −80 °C. Mice were euthanized by isoflurane overdose and death was confirmed by cervical dislocation followed by perfusion with sterile DPBS. The right inferior lobe of the lung and the left hemisphere of the brain or trachea were harvested. A section of each tissue was weighed and homogenized in 500 μL DMEM with a sterile 5 mm glass bead (Fisher Scientific, Emeryville, CA, USA) at 30 Hz for 4 min using a TissueLyser II (Qiagen, Germantown, MD, USA) automated homogenizer. Homogenates were cleared by centrifugation at 10,000× *g* for 4 min and the clarified supernatant was stored at −80 °C for up to 2 weeks before analysis. The remaining lung was inflated by 10% formalin injection and preserved together with the other tissues in 10% formalin for 2–3 days and then transferred to 70% ethanol. Tissues were then embedded in paraffin and cut at 5 μm thick sections before being stained with hematoxylin and eosin for evaluation.

### 2.5. SARS-CoV-2 Plaque Assays

Fluid collected from oropharyngeal swabs, residual inocula, and clarified supernatant from lung, trachea, and brain homogenates were thawed once at room temperature and assayed to quantify infectious SARS-CoV-2. Undiluted (125 μL) and serially 10-fold-diluted samples in DMEM containing 1% FBS were inoculated onto confluent monolayered Vero-E6 or Vero-E6-*TMPRSS2*-T2A-*ACE2* (for omicron only) cells in 12-well cluster plates and incubated for 1 h at 5% CO_2_ in a humidified 37 °C chamber. After the incubation, cell monolayers were overlaid with 0.5% agarose dissolved in DMEM with 5% FBS and 1× antibiotic–antimycotic and held for 2 (omicron only) or 3 (all other variants) days at 5% CO_2_ and 37 °C in a humidified incubator. After 2 or 3 days, cells were fixed for >30 min with 4% formaldehyde and then agarose plugs were removed. Cells were stained with 0.05% crystal violet in 20% ethanol for 10 min then rinsed 3 times with water. Plates were inverted to dry completely and the number of plaques in each well was counted. Viral titers were recorded as the reciprocal of the highest dilution where plaques were noted and are represented as PFU per mL of inoculum or swab liquid, or per mg of tissue. Each value reported represents a titer calculated from 1 technical replicate titration of serial dilutions. The lower limit of detection (LOD) of the assay was 8 PFU/mL. Samples with no detectable plaques are reported under the LOD line on graphs.

### 2.6. Histopathological Analyses

A board-certified veterinary pathologist reviewed all formalin-fixed, paraffin-embedded, hematoxylin- and eosin-stained lung and brain tissues in this study. Either the right half of the brain or the right half and anterior quarter of the left half of the brain were submitted for evaluation. The cerebrum, cerebellum, and medulla were all represented. The pathologist was masked to treatment groups prior to initial slide evaluation and was unmasked for a second round of scoring to account for baseline lesions in DPBS mice. A total of 11–13 mice inoculated with each variant at 4 and 6 dpi (5–7 mice on each day) were evaluated. For each mouse, 1 slide containing lung tissue and 1 slide containing brain tissue were used for scoring. A modified version of our previously developed histopathologic grading scheme was used to evaluate SARS-CoV-2 pulmonary lesions [22]. Briefly, severity scores range from 0 (no significant findings) to 4 based on the percentage of affected parenchyma and the severity of interstitial pneumonia, with an additional point possible for a high (>25%) neutrophilic component, necrotizing vasculitis, and/or microthrombi, for a maximum possible score of 7. Histopathologic grading criteria for the brain were developed for this study. Given that neurologic lesions were multifocally distributed, scores were based on the presence of specific lesions. Leptomeningeal and/or perivascular lymphocytic infiltrates, neutrophilic infiltrates, gliosis, and neuronal necrosis each contributed 1 point for a maximum possible score of 4.

### 2.7. Immunofluorescence

A total of 2–3 mice per variant with lung or brain titers above the LOD at 2, 4, and 6 dpi were evaluated by an unblinded operator. For each mouse, 3 regions of each lung and brain were randomly selected for immunofluorescence analyses. After 48 h of formalin fixation, the lung and right hemisphere of the brain were placed in 0.45 M EDTA in PBS for 3 days. The tissues were cryoprotected in 30% sucrose (weight/volume) in PBS overnight and embedded in an optimal cutting temperature compound (Scigen Scientific Gardena, Houston, TX, USA). Tissue sections of the lung and cortical regions of the brain were obtained at 14 μm in thickness and stored at −20 °C until use. Immunostaining was performed as described previously [35]. The primary antibody dilutions and detection methods are shown in Table 2. Briefly, sections were blocked with 5% horse serum at room temperature for one hour before the application of primary antibodies that were incubated at 4 °C overnight. Signal amplifications were performed as described in Table 2. Direct secondary antibodies were incubated at room temperature for 1 h, and an additional 30 min at room temperature if further amplification was used. DAPI was used for nuclei counterstaining. For RNAscope in situ hybridization, probes are as listed as V-nCoV2019-S #848561-C1, Mouse IL-1B #316891, Mouse IL-6 Cat. #315891-C2, Mouse CCL5 Cat. #469601-C2, Mouse Aif1 (Iba1) Cat. #319141-C3, and V-nCoV2019-S Cat. #848561-C3 (Advanced Cell Diagnostics, Newark, CA, USA), and the manufacturer’s protocol was used. Images were acquired using an Olympus FV3000 confocal microscope (Olympus Corporation, Tokyo, Japan).

### 2.8. Statistical Analyses

Data from all mice in the study are included in analyses. Statistical assessments were performed using GraphPad Prism version 10.4.1 (Boston, MA, USA). Main-effect two-way ANOVA tests were performed on mouse weights normalized to day-0 values just prior to virus inoculation and multiple comparisons were performed with Dunnett’s test. Two-way ANOVA tests were performed on log_10_ transformed SARS-CoV-2 titers in different tissues and multiple comparisons were computed according to Tukey’s method. Histology scores were analyzed using the “stats” [36] and “multicomp” [37] packages in R. Linear regression was used to analyze lung histology scores in K18 and C57BL/6J mice with virus variant and dpi as interacting variables. Contrast matrices were used to conduct pairwise comparisons between all variants at 4 and 6 dpi, as well as for each variant between 4 and 6 dpi. Due to small sample sizes and the high number of zero scores, histologic scores for brain lesions were compared between viral variants using a nonparametric Mann–Whitney U test. This test was applied for each pair of variants at each time point, as well as between time points for variants with data available for both 4 and 6 dpi.

## 3. Results

### 3.1. SARS-CoV-2 Variants Infect Oropharynx, Lungs, and Brains of K18 Mice

**Figure 1:**
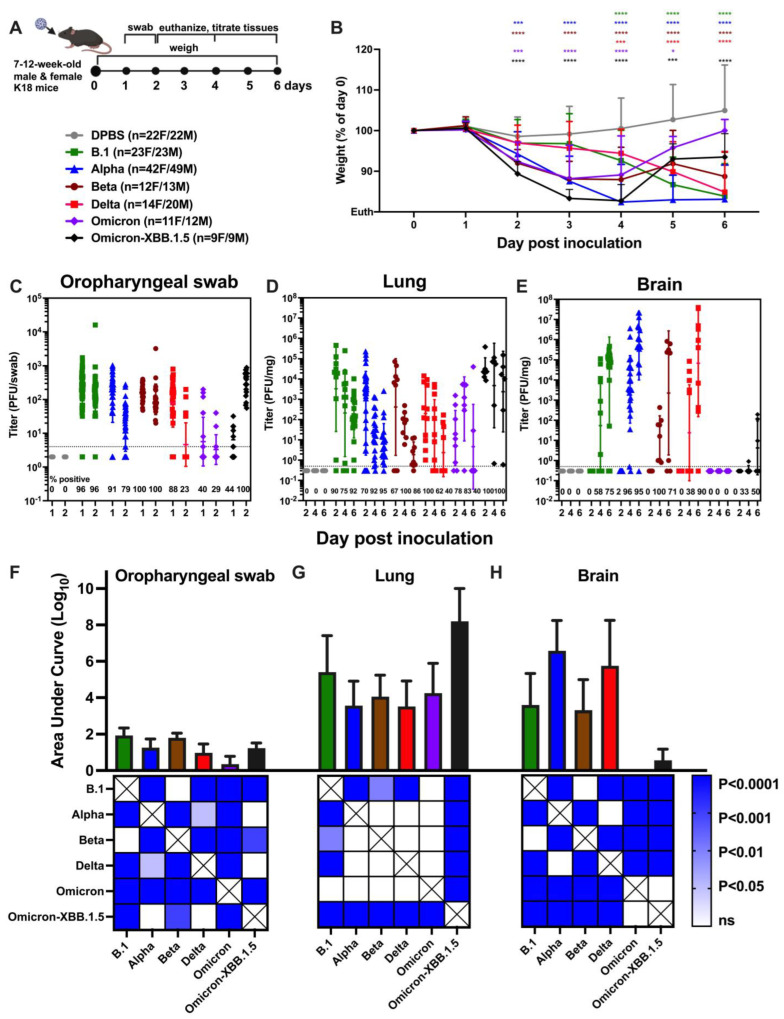
SARS-CoV-2 variants produce varying degrees of weight loss and infectious virus in the oropharynx, lung, and brain of K18 mice. (**A**) Experimental design: Seven to–12-week-old male and female mice of approximate equal sex ratio were inoculated intranasally with DPBS (mock) or 10^4^ PFU of different SARS-CoV-2 variants. Mice were monitored for disease signs and weighed daily, and the oropharyngeal cavity was swabbed on 1 and 2 days post inoculation (dpi). Subsets of mice were euthanized at 2, 4, and 6 dpi. (**B**) Mean weight change represented as a percentage of weight of each mouse on 0 dpi, prior to inoculation. Error bars show standard deviations. Colored symbols above weight graphs match virus groups and show significant differences in mean body weight compared to mock (2-way ANOVA), where *p* < 0.05 is shown as *; 0.001 as ***; and 0.0001 as ****. Infectious SARS-CoV-2 titrated from (**C**) oropharyngeal swabs collected 1 and 2 dpi, and from necropsy samples in (**D**) lung and (**E**) brain on 2, 4, and 6 dpi, quantified by plaque assays. Symbols represent measurements from individual animals, horizontal lines show the geometric mean, and error bars represent the geometric standard deviation. Numbers below the dotted limit-of-detection line indicate the percentage of mice with a detectable SARS-CoV-2 titer. The limit of detection of the assays was 4 PFU/swab for oropharyngeal swab and 0.5 PFU/mg for lung and brain. (**F**) SARS-CoV-2 infection kinetics expressed as area under the infection curve (AUC) in oropharyngeal swabs, (**G**) lungs and (**H**) brains. Colors in the squares underneath panels (**F**–**H**) show differences in mean AUC by strain analyzed using one-way ANOVA tests, where the darker the color, the smaller the *p* value. Each sample was titrated once in 3–6 serial 10-fold dilutions. Mice were from different replicate experiments as follows: DPBS: 11 experiments; B.1: 6 experiments; B.1.1.7 [alpha]: 6 experiments); B.1.351 [beta]: 2 experiments; B.1.617.2 [delta]: 4 experiments; B.1.1.529 [omicron]: 2 experiments; B.1.1.529-XBB.1.5 [omicron-XBB.1.5]: 1 experiment. The infographic in A was generated using Biorender 2025.

In the present study, we performed a comprehensive retrospective analysis comparing SARS-CoV-2 variant infection dynamics in K18 mice using strains of B.1, alpha (B.1.1.7), beta (B.1.351), delta (B.1.617.2), omicron (B.1.1.529), and omicron-XBB.1.5 (Table 1), or DPBS as a control. Male and female K18 mice aged 7–12 weeks in groups ranging from 18 to 91 animals were inoculated intranasally (i.n.) with DPBS or 1 × 10^4^ plaque-forming unit (PFU) SARS-CoV-2. The animals in this analysis served primarily as controls for efficacy studies examining different candidate therapeutics, and the group size variability is a result of differences in individual study designs. Oropharyngeal swabs were collected 1 and 2 dpi; body weight was monitored daily to 6 dpi; and subsets of mice were euthanized 2, 4, or 6 dpi (Figure 1A).

All SARS-CoV-2 variants produced weight loss in K18 mice (Figure 1B) and led to moribundity or ataxia by 5–6 dpi, with most animals in B.1, alpha, beta, and delta groups requiring euthanasia prior to defined study endpoints. The variants showed different kinetics of weight loss compared to DPBS (statistical designations on Figure 1B) and each other (Supplementary Table S1). DPBS mice showed a minor drop in weight 2 dpi, likely due to stress from anesthesia and/or oropharyngeal swabbing. Mice inoculated with alpha, beta, omicron, and omicron-XBB.1.5 exhibited significant weight loss (*p* < 0.00001) 2–6 dpi compared to DPBS. Weight loss for B.1 and delta was delayed and became evident at 4–6 dpi (*p* < 0.0001, all weight assessments used two-way ANOVA). Notably, in contrast to the other variants, the mean body weight of cohorts inoculated with either omicron variant began to recover after 4 dpi, reaching 90–95% of their starting weight by 6 dpi. B.1 infection resulted in less weight loss than alpha at 3 and 4 dpi, beta at 2, 3, and 4 dpi, omicron at 2, 3, 5 and 6 dpi, and omicron-XBB.1.5 at 2, 3 4 and 6 dpi. Alpha produced more weight loss than beta at 4 and 5 dpi, omicron at 4–6 dpi, and omicron-XBB.1.5 at 5 and 6 dpi. Beta caused greater weight loss than omicron at 2 and 3 dpi and delta and omicron-XBB.1.5 on 2, 3 and 4 dpi. Omicron-XBB.1.5 caused greater weight loss than omicron at 4 dpi. In summary, all four non-omicron SARS-CoV-2 variants induced weight loss at levels that met euthanasia criteria in K18 mice between 2 and 6 dpi, with a 2-day delay in weight loss for B.1 and delta compared to alpha, beta, and omicron. Weight recovery was only observed in mice inoculated with omicron variants, none of which met euthanasia criteria.

To examine SARS-CoV-2 in the upper respiratory tract, virus titers were measured from oropharyngeal swabs using plaque assays (Figure 1C) and compared statistically by day (Supplementary Table S2) and variant (Supplementary Table S3). SARS-CoV-2 was detected in swabs from the majority (79–100%) of K18 mice inoculated with B.1, alpha, beta, or delta at 1 dpi, and from B.1, alpha, or beta mice at 2 dpi. In contrast, virus detection in swabs was less frequent (40–44%) at 1 dpi in mice inoculated with either omicron variant, although all omicron-XBB.1.5 mice had detectable virus in their swabs at 2 dpi. Titers in mice infected with alpha and delta were significant higher at 1 compared to 2 dpi. In contrast, omicron-XBB.1.5-inoculated mice shed significantly higher levels of virus in swabs at 2 compared to 1 dpi. When comparing all variants 1 dpi, mice inoculated with B.1, alpha, beta, and delta had significantly higher mean swab titers than those inoculated with omicron and omicron-XBB.1.5. At 2 dpi, B.1, beta, and omicron-XBB.1.5 groups had similar mean titers, which were significantly higher than those in alpha, delta, and omicron groups. The highest mean titers were observed in swabs from B.1-infected mice, followed by beta, alpha, delta, and finally omicron. Area under curve (AUC) analysis (Figure 1F) showed that oral shedding was significantly higher in B.1 and beta than alpha, delta, and omicron-XBB.1.5 groups on both days, while omicron showed significantly lower shedding. These data demonstrate that all SARS-CoV-2 variants were shed in the upper respiratory tract of K18 mice and that omicron produced lower titers compared to other variants and omicron-XBB.1.5. Further, omicron-XBB.1.5 exhibited delayed kinetics, with higher titers at 2 versus 1 dpi, which contrasts with all the other variants where mean titers at 1 dpi were higher than at 2 dpi.

To examine infection dynamics in the lung, subsets of mice were euthanized at 2, 4, and 6 dpi, and SARS-CoV-2 levels in the inferior lung lobe were measured by titration (Figure 1D) and compared statistically by day (Supplementary Table S2) and variant (Supplementary Table S3). Infectious virus was detected in the lungs of most SARS-CoV-2-inoculated K18 mice at 2, 4, or 6 dpi, with titers ranging from the limit of detection (0.5 PFU/mg) to over 10^5^ PFU/mg. Mean lung titers for alpha, beta and delta decreased from 2 to 6 dpi while mean titers for B.1 and both omicron variants remained consistent across all time points. When comparing mean lung titers between variants, B.1 showed significantly higher titers than omicron; alpha had lower titers than omicron-XBB.1.5; and omicron-XBB.1.5 had higher titers than omicron at 2 dpi. At 4 dpi, mean lung titers of omicron-XBB.1.5 were significantly higher than for alpha and delta. By 6 dpi, lung titers for omicron-XBB.1.5 were significantly higher than those for alpha, beta, and delta. Analysis of lung titers by AUC (Figure 1G) showed that omicron-XBB.1.5 had significantly higher levels than alpha, beta, delta, and omicron. These results demonstrate that all variants infect the lung of K18 mice 2–6 dpi, with higher mean titers for omicron-XBB.1.5 compared to most of the other variants.

Next, we evaluated SARS-CoV-2 infection in the brain of K18 mice (Figure 1E) and compared mean titers statistically by day (Supplementary Table S2) and variant (Supplementary Table S3). Infection rates in the brain varied more than in lungs, with titers ranging from the limit of detection (0.5 PFU/mg) to over 10^7^ PFU/mg. Most variants produced detectable brain titers in some mice at 4 or 6 dpi, but never at 2 dpi. In contrast, omicron (B.1.1.529) remained undetectable in the brain at all evaluated times. Brain infection was detected in only 33 or 50% of omicron-XBB.1.5 mice at 4 or 6 dpi, respectively. For B.1, alpha, and beta, mean brain titers increased significantly from 4 to 6 dpi. When comparing mean titers across different variants by day, alpha showed significantly higher titers than B.1, beta, and omicron-XBB.1.5 at 4 dpi. At 6 dpi, B.1, alpha, and delta showed significantly higher titers than omicron-XBB.1.5. AUC analysis (Figure 1H) for all time points also revealed significantly higher brain titers for alpha and delta compared to B.1, beta, omicron, and omicron-XBB.1.5. These results indicate that the B.1, alpha, beta, and delta variants are more neuroinvasive and produce higher brain titers in K18 mice compared to the omicron variants.

### 3.2. Severity of Histopathologic Changes in the Lung and Brain of SARS-CoV-2-Infected K18 Mice Is Variant-Dependent

**Figure 2:**
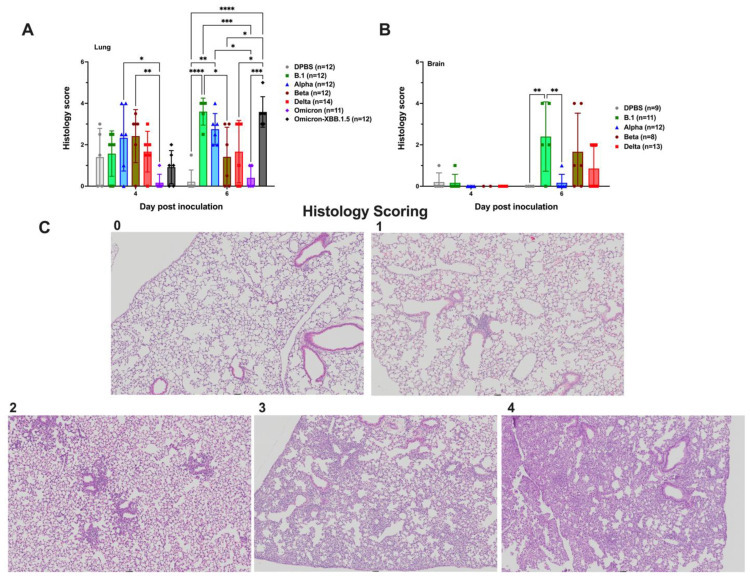
SARS-CoV-2 variants produce varied levels of histopathological changes in the lung and brain of K18 mice. Mean histology scores in (**A**) lung and (**B**) brain 4 and 6 dpi. Differences in histology scores across groups were analyzed by 2-way ANOVA (lungs) and nonparametric Mann–Whitney U tests (brain); only significant differences are shown, with *p* < 0.05 as *; <0.01 as **; <0.001 as ***; <0.0001 as ****. (**C**) Pulmonary lesions for all SARS-CoV-2 variants in affected mice were consistent with interstitial pneumonia and characterized by decreasing air space, increasing cellularity, and increasing thickness of alveolar septa resulting in histologic scores ranging from 1 to 4.

To assess the pathogenicity of SARS-CoV-2 infection, histopathological changes in the lung (Figure 2A) and brain (Figure 2B) were evaluated in subsets of 5–7 K18 mice euthanized at 4 or 6 dpi. Each mouse lung was quantitatively scored histologically using a four-point base severity scale (Figure 2C), with up to three additional points possible for unique features such as >25% neutrophilic inflammatory component, necrotizing vasculitis, and/or microthrombi. In the lung, histology scores ranged from 0 to 4 at 4 dpi and from 0 to 5 at 6 dpi. DPBS-treated mice exhibited scores ranging from 0 to 3 at 4 dpi and 0 to 1 at 6 dpi, likely reflecting pulmonary inflammation due to intranasal inoculation of DPBS. Mice infected with omicron showed lower lung scores compared to alpha, beta, and delta variants at 4 dpi (*p* < 0.05–0.001, two-way ANOVA), and lower lung scores compared to all other variants at 6 dpi (*p* < 0.05–0.0001, two-way ANOVA). The mean scores for omicron-infected mice were not statistically different from those in the DPBS group (*p* > 0.05, two-way ANOVA). Only the omicron-XBB.1.5 mice exhibited a significant increase in lung score from 4 to 6 dpi (*p* ≤ 0.05, two-way ANOVA).

For histologic evaluation of brain tissues, a grading scheme was developed and used. The scheme assigned one point each for leptomeningeal and/or perivascular lymphocytic infiltrates, neutrophilic infiltrates, gliosis, and neuronal necrosis, for a maximum possible score of four. Neither of the omicron variants were included in the brain histology analyses due to the absence of infectious virus in that tissue. At 4 dpi, brain histology scores for mice infected with any variant (excluding omicron) ranged from 0 to 1, with no significant differences compared to DPBS-treated mice (*p* > 0.05, Mann–Whitney U). At 6 dpi, brain scores for B.1-, beta-, and delta-infected mice were significantly higher than at 4 dpi (*p* ≤ 0.05, Mann–Whitney U). There was no change in brain scores for alpha or DPBS mice between 4 and 6 dpi (*p* > 0.05, Mann–Whitney U). Only 1 out of 12 of alpha-infected mice developed brain lesions, despite having high brain titers (Figure 1E). At 6 dpi, B.1-infected mice showed significantly higher brain histology scores than alpha and DPBS groups (*p* ≤ 0.05, Mann–Whitney U).

**Figure 3:**
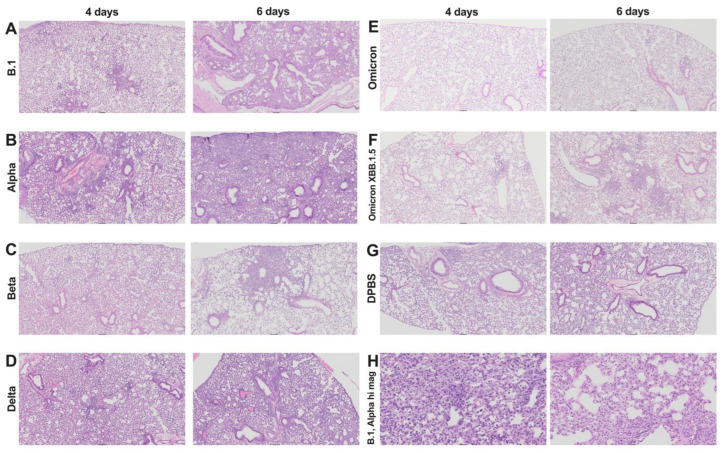
Representative images of histopathological features in lungs of K18 mice infected with SARS-CoV-2 variants at 4 and 6 dpi illustrating a spectrum of presentations ranging from no lesions to severe inflammation. Images were chosen based on the mean histologic score for each variant/day combination. Images in A-G show H&E-stained lung at 5× magnification. Key features at this magnification include loss of alveolar (clear) space, perivascular and/or peribronchiolar mononuclear aggregates, and interstitial and/or alveolar cellular infiltrates. (**A**) B.1, 4 dpi (score 2); 6 dpi (score 4). (**B**) Alpha, 4 dpi (score 2.5) and 6 dpi (score 4). (**C**) Beta, 4 dpi (score 3) and 6 dpi (score 3). (**D**) Delta, 4 dpi (score 1.5) and 6 dpi (score 3). (**E**) Omicron, 4 dpi (score 0) and 6 dpi (score 1). (**F**) Omicron-XBB.1.5, 4 dpi (score 1) and 6 dpi (score 3). (**G**) DPBS, 4 dpi (score 1.5) and 6 dpi (score 0). (**H**) Higher-magnification (20×) lung histologic score 4 at 4 dpi (B.1) and 6 dpi (alpha). Inflammation transitions from neutrophilic (4 dpi) to histiocytic (6 dpi).

Lung lesions in all K18 mice were consistent with interstitial pneumonia (Figure 3A–F,H). These lesions were characterized by thickened alveolar septa, increased cellularity, peribronchiolar and/or perivascular leukocytic aggregates, and loss of alveolar space, which was milder but not absent in DPBS mice (Figure 3G), where some inflammation likely stemmed from inoculation of DPBS only. At 4 dpi, inflammation was a mix of lymphoplasmacytic to neutrophilic, with occasional reactive endothelial cells and rare vasculitis. By 6 dpi, inflammation was primarily composed of histiocytes, with variable numbers of lymphocytes and plasma cells. Evidence of injury repair, including type II pneumocyte hyperplasia, was frequently observed.

**Figure 4:**
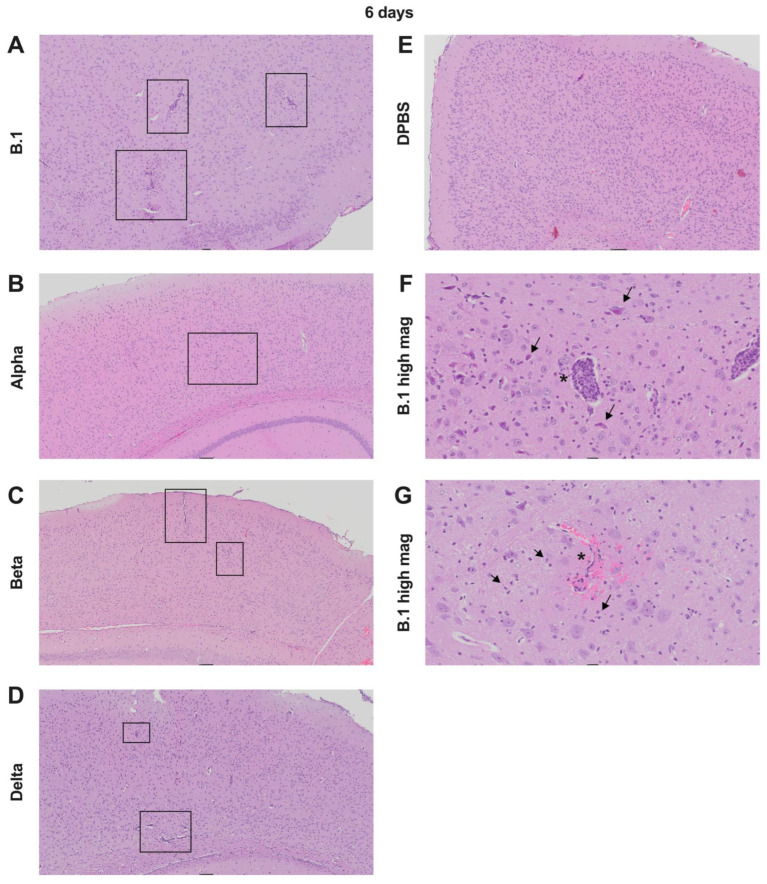
Representative histopathology of affected brain tissues in K18 mice infected with different SARS-CoV-2 variants at 6 dpi. Images in A-E are H&E-stained cortex at 5× magnification. Boxes highlight affected regions of inflammation. Key lesions at this magnification include perivascular lymphoplasmacytic cuffs and gliosis (increased cellularity). (**A**) B.1 (score 4); (**B**) alpha (score 1); (**C**) beta (score 4); (**D**) delta (score 2); (**E**) DPBS (score 0). (**F**,**G**): B.1 (score 4) key lesions at 20× magnification from separate sections shown in other panels. (**F**) Neuronal necrosis (arrows) and perivascular cuffing (asterisk) in a mouse infected with B.1 at 6 dpi. (**G**) Neutrophilic infiltrates (arrows) and fibrin thrombus with microhemorrhage (asterisk) in a mouse infected with B.1 at 6 dpi.

At 6 dpi, brain lesions in B.1-, alpha-, beta-, and delta-infected mice (Figure 4A–D) demonstrated perivascular lymphoplasmacytic cuffs and gliosis, which was absent in the DPBS group (Figure 4E). Neutrophil infiltration (Figure 4F) and fibrin thrombi with microhemorrhages (Figure 4G) were also observed in the brains of some variant-infected mice.

Together, our data show that SAR-CoV-2 infection results in variable pathological and inflammatory changes in the lung and brain of K18 mice, ranging from mild to severe, depending on the variant. Compared to other variants, omicron produced the least histopathologic lesions. The low histologic lung scores in DPBS-treated mice suggest that some of the inflammation may stem from the intranasal administration of liquid.

### 3.3. SARS-CoV-2 Variants Infect Oropharynx, Trachea, and Lungs of C57BL/6J Mice but Do Not Exhibit Neurotropism

**Figure 5:**
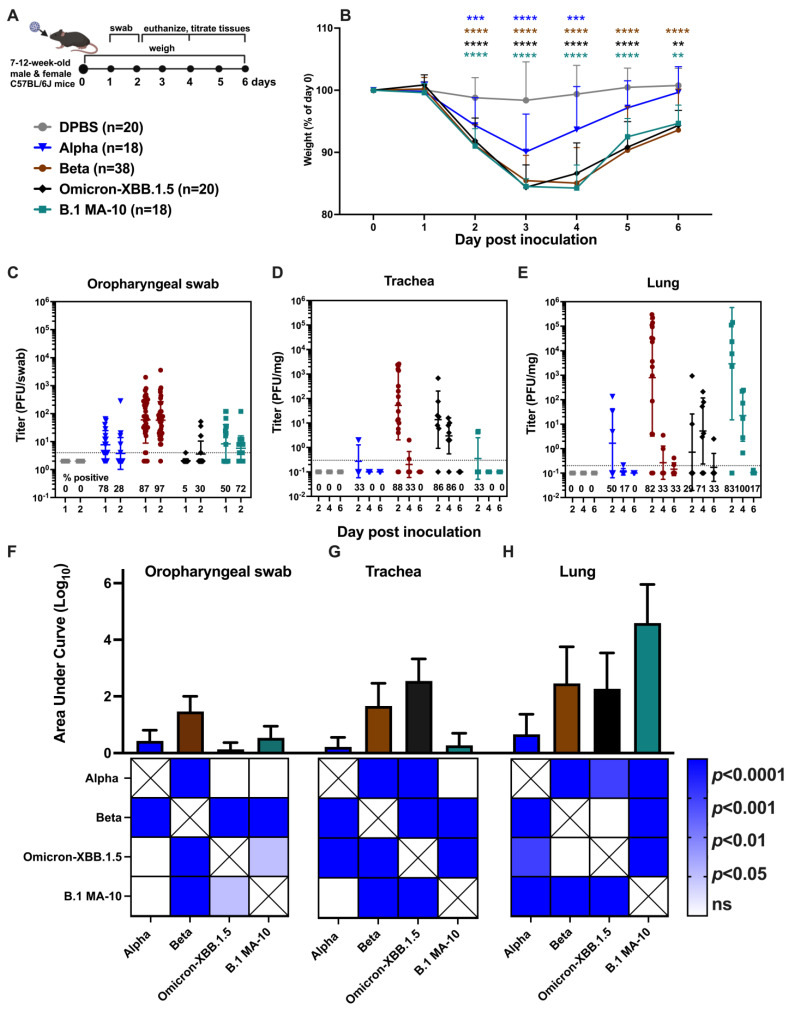
SARS-CoV-2 variants produce varying degrees of weight loss and infectious virus in the oropharynx, trachea, and lung of C57BL/6J mice. (**A**) Experimental design: Seven to12-week-old male and female mice of equal sex ratio were inoculated intranasally with DPBS or 10^5^ PFU of alpha, beta, omicron-XBB.1.5, or 10^4^ PFU of mouse-adapted B.1 (B.1 MA-10) SARS-CoV-2. (**B**) Mean weight change represented as a percentage of weight of each mouse on 0 dpi, prior to inoculation. Error bars show standard deviations. Colored symbols above weight graphs match virus groups and show significant differences in mean body weight compared to mock (2-way ANOVA), where *p* < 0.01 is shown as **; 0.001 as ***; and 0.0001 as ****. Infectious SARS-CoV-2 titrated from (**C**) oropharyngeal swabs collected 1 and 2 dpi, and (**D**) trachea and (**E**) lung on 2, 4, and 6 dpi, as quantified by plaque assay. Each symbol shows measurements from individual animals; the horizontal lines show geometric means, and error bars represent geometric standard deviations. The numbers below the dotted limit-of-detection line indicate the percentage of mice with a detectable SARS-CoV-2 titer. The limit of detection of the assays was 4 PFU/swab and 0.5 PFU/mg for lung and 0.5 PFU/mg brain. (**F**–**H**) SARS-CoV-2 infection kinetics expressed as areas under the infection curve (AUC) in the oropharyngeal swabs, lungs, and brains. Colors in the squares underneath panel **F** show differences in mean AUC by strain analyzed using one-way ANOVA tests, where the darker the color, the smaller the *p* value. Data shown are from 3 replicate experiments. The infographic in **A** was generated using Biorender.

We next evaluated a subset of variants (alpha, beta, and omicron XBB.1.5) in C57BL/6J mice. These variants were selected because they were used as control groups in therapeutic efficacy studies that are not the focus of this paper. We compared variant infection dynamics to mouse-adapted (MA10) SARS-CoV-2 that was generated from serial murine passage of a B.1 strain [28], hereafter referred to as B.1 MA-10, in 7–12-week-old male and female C57BL/6J mice of equal sex ratio (Figure 5A). At least 18 C57BL/6J mice were inoculated i.n. with 1 × 10^5^ PFU alpha, beta, omicron-XBB.1.5, or 1 × 10^4^ PFU B.1 MA-10 and harvested at 2, 4 and 6 dpi. A lower dose of B.1 MA-10 was used to account for its high infectivity in C57BL/6J mice [28]. At 2–4 dpi, all variant-infected groups showed rapid and significant weight loss (8–15% of starting body weight) compared to DPBS-treated mice (*p* < 0.0001, two-way ANOVA) (Figure 5B). Although the weight of all variant groups stabilized and started recovering by 4–6 dpi, only alpha-infected mice regained weight to levels comparable to DPBS-treated mice, with no significant differences at 5 and 6 dpi (*p* > 0.05, alpha vs. DPBS, *p* < 0.001 other variants vs. DPBS, two-way ANOVA). To assess SARS-CoV-2 infection in the respiratory tract and brain, we collected oropharyngeal swabs at 1 and 2 dpi, and harvested the trachea, lung, and brain at 2, 4, and 6 dpi for plaque assays. No infectious virus was detected in the brains of any C57BL/6J mice. At 1 and 2 dpi, between 50 and 97% of mice infected with alpha, beta, and B.1 MA-10 had detectable virus in oropharyngeal swabs. In contrast, omicron-XBB.1.5 was detected in only 5% of mice at 1 dpi and 30% at 2 dpi (Figure 5C). Beta-infected mice had significantly higher mean titers in swabs compared to all other variants and DPBS (*p* < 0.00001, two-way ANOVA). No significant differences in mean titers were detected between 1 and 2 dpi for any variant (Supplementary Table S4). AUC analysis (Figure 5F) showed significantly higher cumulative swab virus levels in beta-infected mice compared to omicron-XBB.1.5 or B.1 MA-10.

In the trachea, SARS-CoV-2 was most frequently detected in C57BL/6J mice at 2 dpi, with detection rates ranging from 33 to 88% of the cohort (Figure 5D). B.1 MA-10 showed detection in only two of six mice at 2 dpi. By 4 dpi, beta-infected mice showed significantly lower tracheal virus levels compared to 2 dpi, while omicron-XBB.1.5 levels peaked a 2 dpi and then declined significantly by 4 and 6 dpi (Supplementary Table S4). AUC analysis (Figure 5G) showed significantly higher trachea virus levels in beta and omicron-XBB.1.5 compared to alpha and B.1 MA-10 mice.

Lung titers in C57BL6J mice (Figure 5E) were generally higher than those observed in the oropharyngeal swabs or trachea, with some mice reaching titers of 10^6^ PFU/mg. Beta- and B.1 MA-10-infected mice had significantly higher mean lung titers at 2 dpi compared to alpha, omicron-XBB.1.5, or DPBS at 2 dpi. Lung titers for beta-infected mice were significantly lower at 4 and 6 dpi compared to 2 dpi (Supplementary Table S5). In contrast, lung titers in omicron-XBB.1.5-infected mice remained consistent 2, 4, and 6 dpi. AUC analysis (Figure 5H) indicated that B.1 MA-10 produced significantly higher lung titers compared to alpha, beta, and omicron-XBB.1.5, and that beta and omicron-XBB.1.5 had higher titers than alpha.

These results demonstrate distinct patterns of weight loss and tissue viral titers in C57BL/6J mice infected with different SARS-CoV-2 variants, with no variants causing the severe weight loss (20% of starting body weight) that necessitated euthanasia, as seen in K18 mice. Alpha infection resulted in less pronounced weight loss and lower tissue titers compared to other variants. Beta, omicron-XBB.1.5, and B.1 MA-10 exhibited similar tropism and tissue titers, with minor variation across days and strains.

### 3.4. SARS-CoV2 Infection in C57BL/6J Mice Produces Mild Histopathologic Lesions in the Lung

**Figure 6:**
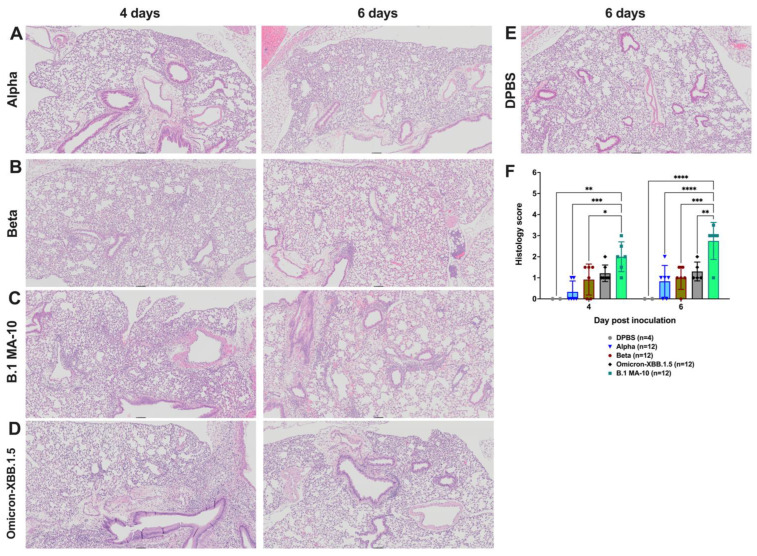
SARS-CoV-2 variants produce varied levels of pathological changes in the lungs of C57BL/6J mice. Images were chosen based on the mean histologic score for each variant/day combination. Pulmonary lesions for all SARS-CoV-2 variants in affected mice are consistent with interstitial pneumonia manifesting as decreasing air space, increasing cellularity, and increasing thickness of alveolar septa that produced histologic scores ranging from 1 to 4. All images are taken at 5× magnification. Key features at this magnification include loss of alveolar (clear) space, perivascular and/or peribronchiolar mononuclear aggregates, and multifocal to diffuse interstitial cellular infiltrates. (**A**) Alpha at 4 dpi (score 1) and 6 dpi (score 1). (**B**) Beta at 4 dpi (score 1.5) and 6 dpi (score 1.5). (**C**) B.1 MA-10 at 4 dpi (score 2) and 6 dpi (score 3). (**D**) Omicron-XBB.1.5 at 4 dpi (score 2) and 6 dpi (score 2). (**E**) DPBS at 6 dpi (score 0). (**F**) Lung histology scores. Differences in histologic scores were analyzed by 2-way ANOVA, *p* < 0.05 as *; <0.01 as **; <0.001 as ***; <0.0001 as ****. Assessment of lung histological scores was made from images viewed at 20× magnification.

Although the overall severity of pneumonia was less pronounced in C57BL/6J mice compared to K18 mice, the lesions observed were similar, consisting of interstitial pneumonia characterized by neutrophilic and lymphoplasmacytic inflammation at 4 dpi and histiocytic and lymphoplasmacytic inflammation at 6 dpi present in milder form in DPBS mice (Figure 6A–E). We used the histology scoring system to evaluate lung lesions in a subset of six C57BL/6J mice per variant, euthanized at 4 or 6 dpi (only two mice per day for DPBS). B.1 MA-10 produced significantly higher lung lesion scores on both days compared to the other variants or DPBS (*p* ≤ 0.05–0.0001) (Figure 6F).

**Figure 7:**
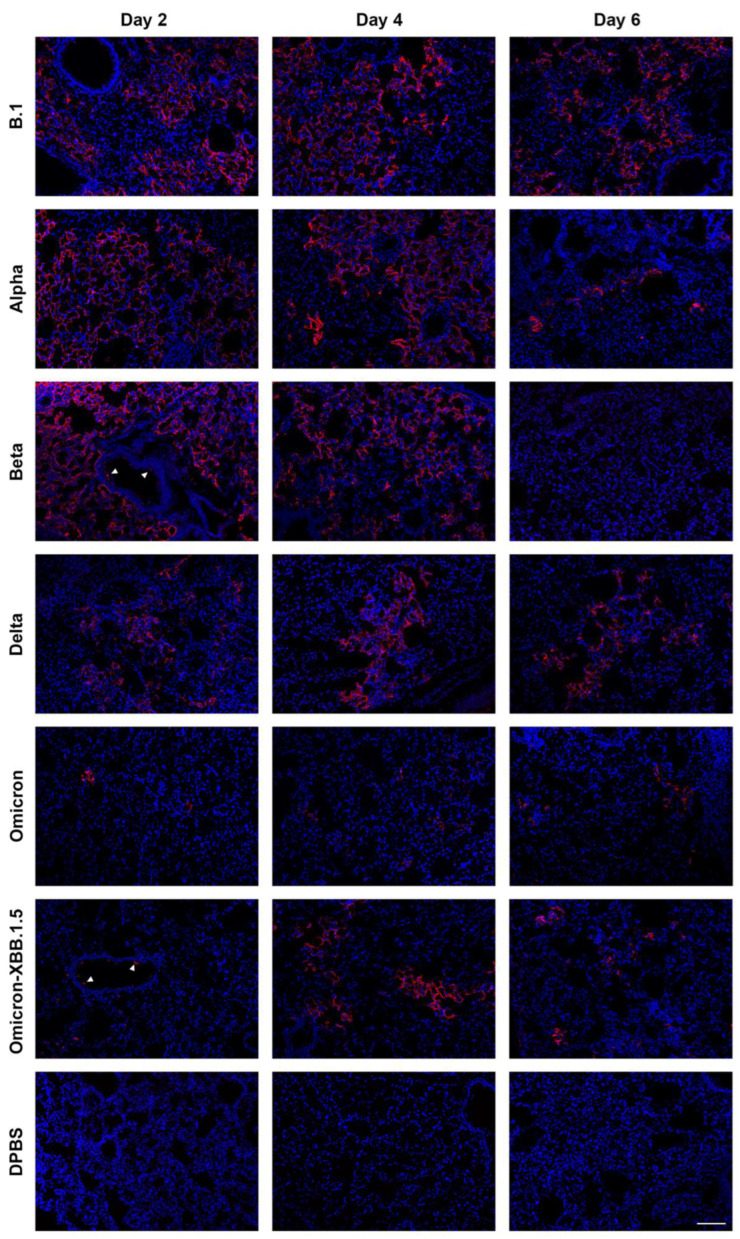
Different SARS-CoV-2 variants show varied levels of infection in the lung of K18 mice at 2, 4 and 6 dpi detected by nucleocapsid protein (NP) immunostaining. Representative NP (red) signal distribution, counterstained by DAPI (blue). Arrowheads indicate rare bronchial epithelial cell infection with SARS-CoV-2. Scale bar shows 100 μm.

**Figure 8:**
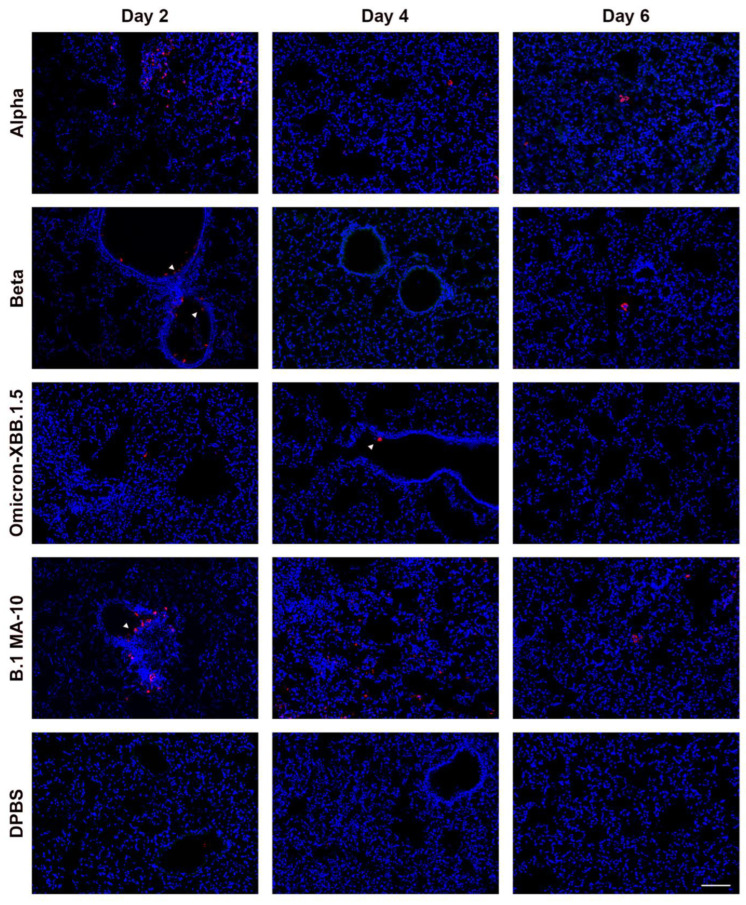
Different SARS-CoV-2 variants show varied levels of infection in the lung of C57BL/6J mice at 2, 4 and 6 dpi detected by nucleocapsid protein (NP) immunostaining. Representative NP (red) signal distribution, counterstained by DAPI (blue). Arrowheads indicate rare bronchial epithelial cell infection with SARS-CoV-2. Scale bar shows 100 μm.

SARS-CoV-2 nucleoprotein staining in the lungs is more extensive in K18 than in C57BL/6J mice. To examine the distribution of SARS-CoV-2 variants in the lungs of K18 (Figure 7) and C57BL/6J (Figure 8) mice at 2, 4, and 6 dpi, we performed immunofluorescence to detect SARS-CoV-2 nucleoprotein (NP) and assessed the distribution of its signal in the tissue. Staining patterns were consistent across mice infected with the same variant, and representative images are shown (Figure 7 and Figure 8). NP-positive cells were detected in both K18 and C57BL/6J mice infected with all evaluated variants in the lung, but not in DPBS-treated mice. K18 mice infected with beta variant exhibited the strongest NP signal at 2 dpi, which decreased by 4 dpi and was absent by 6 dpi. B.1, alpha, and delta variants produced NP staining in the lungs at all time points. Omicron-infected K18 mice showed the weakest NP signal at any time. K18 mice infected with omicron-XBB.1.5 displayed limited NP staining at 2 dpi, peaking at 4 dpi and declining again by 6 dpi. In C57BL/6J mice, NP staining was infrequent but still detectable for all tested variants at 2 and 4 dpi for alpha and beta and 6 dpi for B.1 MA-10. B.1 MA-10-infected C57BL/6J mice showed more NP staining compared to other variants. The morphology and location of the predominant infected cell population along the lining of alveolar septa are consistent with type I and type II pneumocytes. SARS-CoV-2 NP was also occasionally detected in bronchiolar epithelial cells for several variants in both K18 and C57BL/6J mice. Overall, NP was more abundant and widespread for all variants in K18 mice compared to C57BL/6J mice.

**Figure 9:**
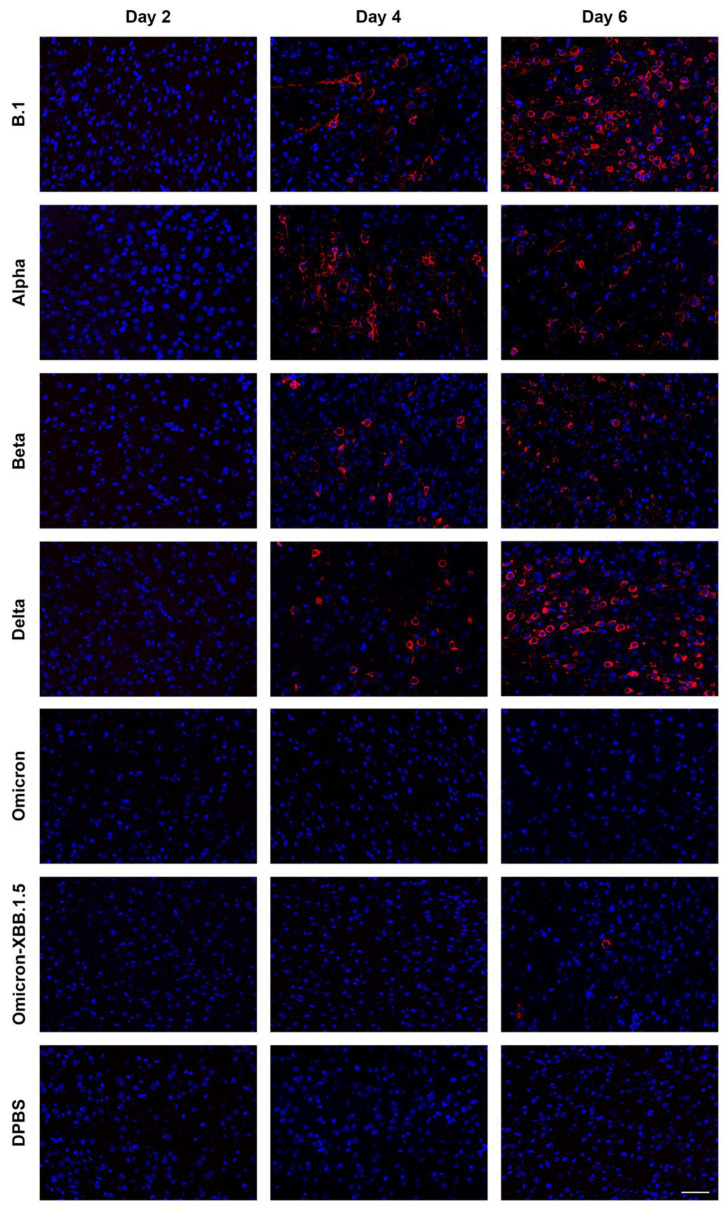
SARS-CoV-2 variants show varied levels of infection in the coronal section of the brain of K18 mice at 2, 4 and 6 dpi detected by nucleocapsid protein (NP) immunostaining. Representative NP (red) signal distribution, counterstained by DAPI (blue), for B.1, alpha, beta, delta, omicron, and omicron-XBB.1.5 at 2, 4 and 6 dpi. Scale bar is 50 μm.

**Figure 10:**
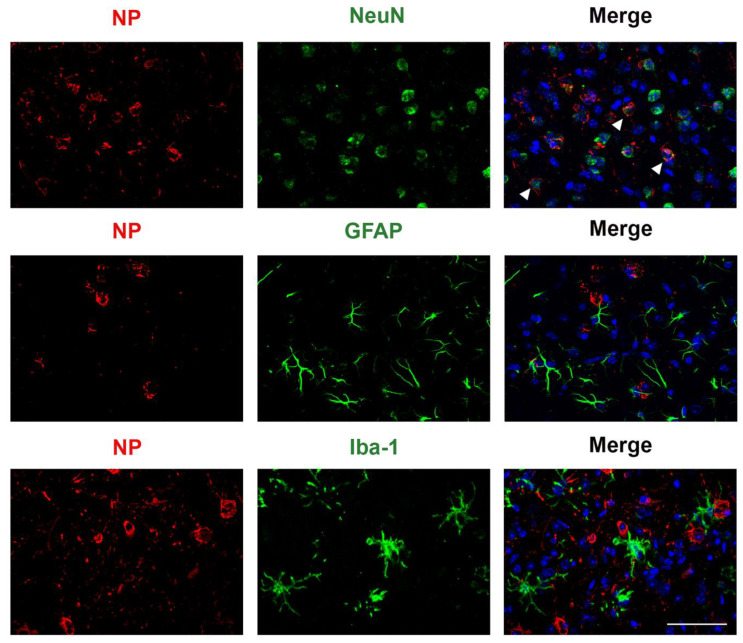
SARS-CoV-2 infection of a K18 mouse with beta shows infections in neurons in the cortex via immunofluorescence at 2 dpi. DAPI-stained nuclei are shown in blue; SARS-CoV-2 NP is in red; NeuN+ neurons, GFAP+ astrocytes, and Iba-1+ microglia are shown in green. Arrowheads show examples of NP+ overlap with neurons. Scale bar is 50 μm.

SARS-CoV-2 nucleoprotein was detected in the cortex of K18 mice infected with B.1, alpha, beta, delta, and omicron-XBB.1.5, but not omicron, and increased from 2 to 6 dpi. To investigate the distribution of SARS-CoV-2 variants in the cortex of K18 mice (Figure 9) at 2, 4, and 6 dpi, we performed immunofluorescence to detect NP distribution. Staining patterns were consistent across mice infected with the same variant, with representative patterns shown. We also performed similar staining in C57BL/6J mice, but NP signal was not detected in the cortex of any of these mice. In K18 mice, NP-positive cells were detected in all SARS-CoV-2 variant-infected groups, but not in DPBS-treated mice. At 2 dpi, little NP signal was detected in the cortex of K18 mice infected with any variant. By 4 dpi, mice infected with B.1, alpha, beta, and delta variants showed widespread NP staining, which increased by 6 dpi. B.1-infected mice exhibited the most NP-positive cells, followed by delta. Omicron-infected K18 mice showed no NP signal in the cortex, and omicron-XBB.1.5-infected mice displayed few NP-stained cells at 6 dpi. We observed that, consistent with previous observations [38,39], neurons are infected by SARS-CoV-2, astrocytes remain uninfected, and activated microglia are detected (Figure 10).

**Figure 11:**
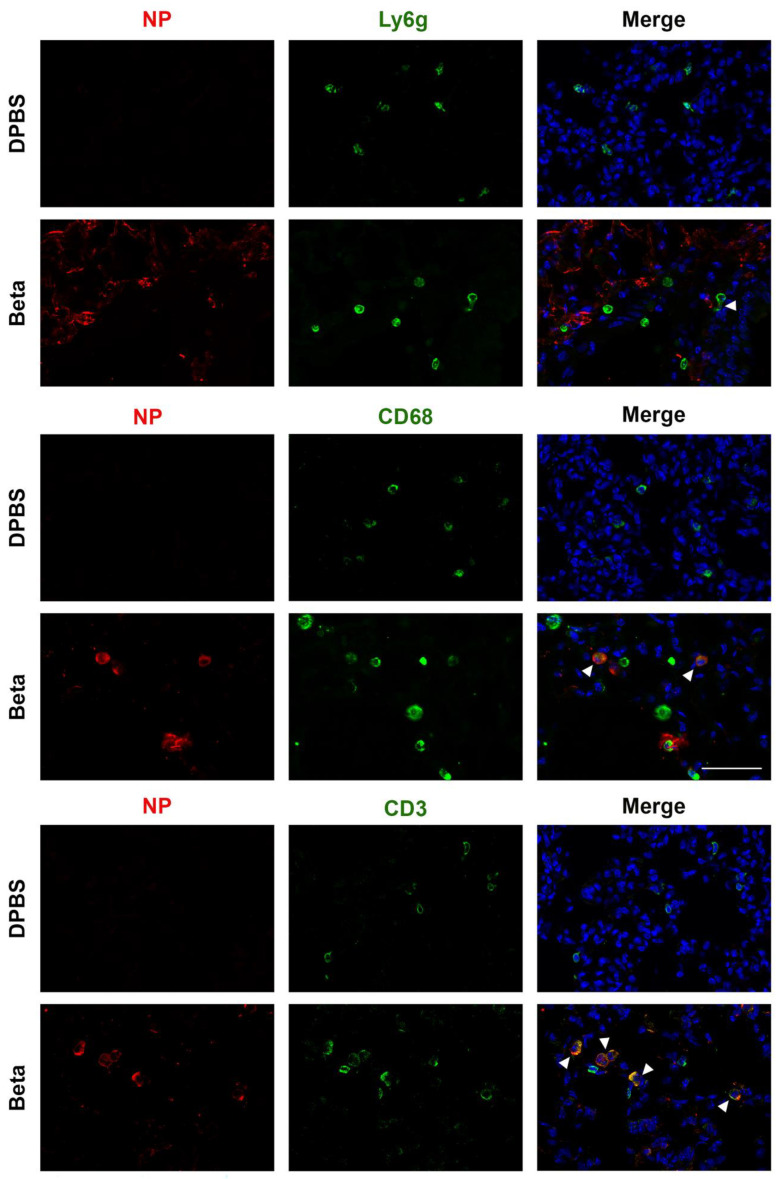
SARS-CoV-2 beta infection of the lung of a K18 mouse produces immune cell markers detected via immunofluorescence at 2 dpi. DAPI-stained nuclei are shown in blue, with SARS-CoV-2 NP in red; Ly6g-positive neutrophils, CD68-positive macrophages, and CD3-positive T cells are detected (green). Arrowheads indicate NP overlap with immune cells. Scale bar is 50 μm.

SARS-CoV-2 beta co-localizes with immune cells and cytokines in the K18 mouse lung. To examine the immune response in the lungs of K18 mice infected with the beta variant at 2 dpi, based on the robust NP distribution, we used RNAscope in situ hybridization to probe for cytokine expression and immunofluorescence to localize immune cells (Figure 11). Staining patterns were consistent across mice, with representative patterns shown. NP staining was observed throughout the lung tissue, with minor patches of non-infected areas. We visualized immune cells including Ly6G+ neutrophils, CD68+ macrophages, and CD3+ T cells, which were also present in DPBS-treated mice. Arrowheads in Figure 11 point to example immune cells containing NP within the cell. We observed NP signals co-localizing with a subset of all immune cell types examined.

**Figure 12:**
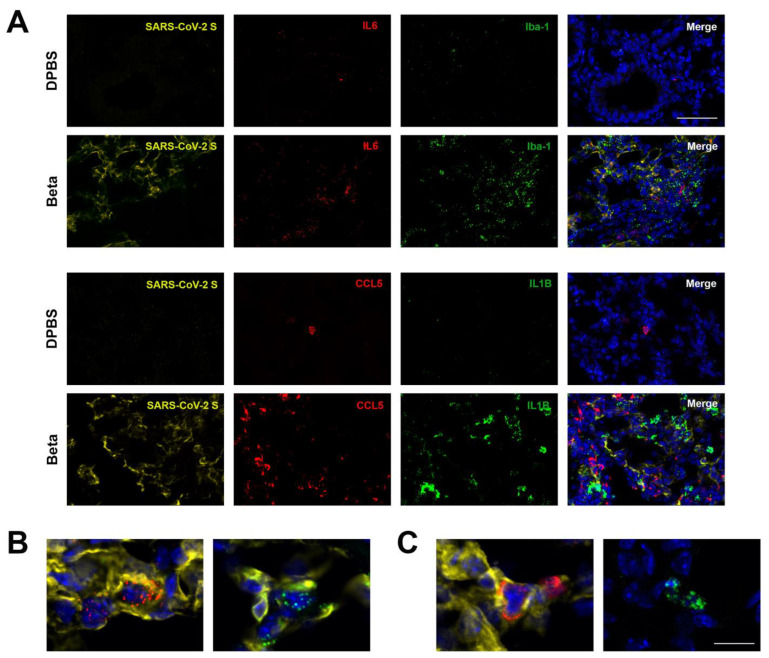
SARS-CoV-2 beta infection of the lung of a mouse shows cytokine and macrophage expression in the lung at 2 dpi. Images were captured using RNAscope via in situ hybridization. (**A**–**C**) SARS-CoV-2 spike (S)-positive cells are detected in yellow, cytokines CCL5 and IL-6 are in red, Iba-1+ macrophages are in green, and IL1β is in green. DAPI-stained nuclei are shown in blue. (**B**) Higher-magnification images of co-localization of signal for IL-6+ and S-positive cells (left) and (right) Iba1+ and S-positive cell. (**B**) Higher-magnification images of co-localization of signal for CCL5+ and S-positive cells (left) and of Il1B+ and S-negative cells (right). Scale bar shows (**A**) 50 μm and (**B**,**C**) 20 μm.

We also used RNAscope to detect cytokine expression. We observed qualitative increases in the cytokines IL-6, IL-1β, and CCL5 in beta-infected K18 mice compared to DPBS-treated mice. CCL5 and IL-6 co-localized with SARS-CoV-2-infected pneumocytes, while IL-1β expression was detected in both infected and uninfected cells, although at a minor level compared to CCL5 and IL-6 and in line with SARS-CoV-2 NP. Iba1+ macrophages were also observed co-localizing with SARS-CoV-2 NP (Figure 12).

## 4. Discussion

Murine models remain essential for studying SARS-CoV-2 virulence and pathogenesis and for evaluating countermeasures against COVID-19. In this study, we used adult male and female K18 and C57BL/6J mice to evaluate disease progression, viral kinetics, tropism, and histopathologic changes for six SARS-CoV-2 variants that previously dominated global circulation. K18 mice express human ACE2, while C57BL/6J mice do not. Although K18 mice represent severe disease and show non-physiological expression of human ACE2 in extrapulmonary tissues including the brain [40], raising concerns about the human relevance of this model and leading us to pursue alternate non-lethal knock-in models [41], K18 mice are still commonly used in SARS-CoV-2 research. Most SARS-CoV-2 variants caused acute weight loss and lethal disease in K18 mice, with recovery observed only in omicron-inoculated mice. All variants were shed in the upper respiratory tract of K18 mice; however, omicron and omicron-XBB.1.5 exhibited lower viral loads in the oropharynx and lungs compared to other variants. Notably, omicron did not invade the brain in any mouse, while omicron-XBB.1.5 produced lower brain titers than other variants in the minority mice where neuroinvasion was detected. Lung tissues of K18 mice displayed variable severity of histopathologic lesions, ranging from multifocal perivascular and peribronchiolar lymphoid aggregates to regionally extensive consolidation and lymphoplasmacytic, histiocytic, and neutrophilic inflammation. Severity of the interstitial pneumonia was variant-specific, similar to findings in other studies [42,43]. The days mice were harvested, variants, and method of histologic grading used in the current study are unique, limiting direct comparison to similar studies. Chen et al. [42] compared lung lesions for alpha, beta, and delta variants in K18 mice exclusively on or prior to 3 dpi. In agreement with their findings, at 4 dpi, the beta variant produced the most severe lesions, followed by alpha and finally delta, though none of these differences were statistically significant. However, by 6 dpi, alpha was associated with the most pronounced changes, while beta had the mildest. Patterns of histopathologic lesions in the lung are consistent with other descriptions of SARS-CoV-2 infection in K18 mice [16,25,42,43], though significant bronchiolar epithelial damage and syncytial cells were not observed as reported in other studies [16,42]. Brain lesions, when present, consisted of multifocal meningitis, gliosis, microthrombi, neutrophilic infiltrates, and rare neuronal necrosis. Neuroinflammation associated with alpha, beta, and delta variants has been demonstrated in other work [43,44] and is supported here. Identified brain lesions are similar to those previously reported, specifically the concentration of inflammatory cells and gliosis around vessels and increasing severity at later times after infection [16,25,43,45]. SARS-CoV-2 nucleoprotein was detected in the brain of K18 mice infected with B.1, alpha, beta, delta, and omicron-XBB.1.5, but not in omicron-infected mice, mirroring the kinetics of infectious virus detection. Omicron infection resulted in the lowest lung histopathology scores, which were comparable to those in DPBS-treated control mice.

In C57BL/6J mice, SARS-CoV-2 variants exhibited distinct patterns of clinical disease, tropism, and viral titers compared to K18 mice. Unlike K18 mice, none of the evaluated variants caused significant irrecoverable weight loss (≥20% of starting weight) requiring euthanasia. Among the variants, alpha caused less pronounced weight loss and had lower viral titers in oropharyngeal swabs, trachea, and lung tissues compared to others. Beta, omicron-XBB.1.5, and B.1 MA-10 displayed similar tropism and tissue titers, with minor inter-day and inter-strain variations. Notably, no virus was detected in the brains of any C57BL/6J mice. Although C57BL/6J mice infected with different variants developed histologic signs of lung inflammation, lesions were less widespread and inflammation was milder than in K18 mice. Consistently, SARS-CoV-2 nucleoprotein staining in lung tissue was more extensive in K18 mice compared to C57BL/6J mice. Both mouse models showed a similar attenuation pattern for omicron compared to earlier variants. Our findings parallel prior studies [19,21,42,43,44,45,46,47,48,49] involving single or multiple SARS-CoV-2 variants. For example, other alpha and delta strains cause severe disease in K18 mice marked by high viral titers, extensive lung damage, systemic inflammation, and elevated mortality. In contrast, C57BL/6J mice generally exhibit mild to moderate disease. However, infection severity can be enhanced through use of mouse-adapted strains. Studies comparing two or more variants show similar patterns to our data: in K18 mice, alpha and beta are more pathogenic than B.1 and delta and other variants are more pathogenic than omicron [19]. The pattern of reduced virulence of omicron compared to earlier variants is consistent with reduced disease in humans [50], mice [46], and hamsters [51,52], and may be mediated by inefficient transmembrane serine protease 2 (TMPRSS2, which facilitates virus entry into host cells) usage in comparison with previous variants [53]. The identification of pneumocytes as a primary target of SARS-CoV-2 are consistent with previous work on K18 mice [16,54] and rhesus macaques [55,56] demonstrating NP in type I and type II pneumocytes by immunohistochemistry. Consistent with previous reports in C57BL/6J mice [27,28], we also observed NP signals in bronchiolar epithelial cells, which is different from the primary alveolar localization in K18 mice [12]. Consistent with other reports [57,58], we observed NP colocalizing with areas of inflammation. The co-localization of NP signal within macrophages and T cells specifically suggests possible infection of these immune cells, which was previously speculated to contribute to potential immune suppression [59]. Our observation of cytokine transcription in the lungs of infected K18 mice are consistent CCL5, IL1β, and IL-6 contributing to the cytokine storm during SARS-CoV-2 infection [60,61]. CCL5 recruits T cells, monocytes, and natural killer cells; IL1β initiates and amplifies inflammation; and IL-6 mediates systemic inflammation [62,63,64].

Our study has several limitations. Experiments were conducted in multiple replicates. While the mice were age-matched and sourced from one commercial vendor and the same person handled inoculation, swabbing, and weighing, we cannot entirely rule out inter-experimental variation as a potential confounder. The use of only three variants in C57BL/6 mice compared to six variants in K18 mice limits direct comparisons of all variants. We also observed discordance between viral titers and histological lesions in some variant groups. For instance, despite high virus levels in the brains of K18 mice for several variants at 4 and 6 dpi, histological lesions were absent at 4 dpi and present only in some individuals at 6 dpi. One possible explanation is that brain lesions or infectious SARS-CoV-2 in tissues was multifocal rather than diffuse, meaning that sectioning at one level of the brain could detect a lesion, while another level might miss it. Similarly, as sections of brain submitted for evaluation were embedded and cut by different technicians, discrepancies in orientation and the amount of tissue examined for each mouse could result in overlooked lesions. Additionally, the delay in lesion development relative to viral titers suggests that neuroinvasion does not always correspond directly to weight loss. This raises the possibility that the absence of weight loss may not necessarily indicate a lack of neuroinvasion. We also observed high variability in histological scores within groups, reflecting significant individual differences in susceptibility and response to SARS-CoV-2 or variability in the amount of virus that reaches the nose or lung despite using the same inoculation dose. This variability and baseline inflammation associated with DPBS inoculation (particularly at 4 dpi) reduced the statistical power to detect differences between viral variants. Increasing sample sizes for histology analyses would enhance the ability to identify subtle, variant-specific pathologic changes more reliably. We also did not identify which cell types contribute to the cytokine expression patterns detected.

Overall, our findings highlight the utility of the lethal K18 model for studying severe SARS-CoV-2 infections and variant-specific virulence, while the non-lethal C57BL/6J model may be better suited for investigating immune responses and multi-variant infection dynamics. The observed differences underscore the importance of selecting the appropriate mouse model based on the specific research question and variant being studied. Additionally, the unique traits of each of the six evaluated SARS-CoV-2 variants emphasize the need for phenotypic characterization of new variants as they emerge.

## Figures and Tables

**Table 1:** SARS-CoV-2 strains used in murine studies. N/A denotes not applicable as this strain was derived from WA1 by serial murine passage.

Strain	Variant	Lineage	Isolation Location	Source, Batch	Passage	Vero Cell Titer (PFU/mL)
hu/USA/CA-CZB-59X002/2020. (MT394529)	B.1	WA1-like	CA, USA	Christopher Miller, UC Davis	p2	2.2 × 10^7^
hu/USA/CA_CDC_5574/2020	alpha	B.1.1.7	CA, USA	BEI Resources, NR-54011, Lot: 70041598	p1	7.7 × 10^6^
hCoV-19/USA/MD-HP01542/2021	beta	B.1.351	MD, USA	BEI Resources, NR-55282, Lot: 70043066	p1	2 × 10^7^
hCoV-19/USA/PHC658/2021	delta	B.1.617.2	TN, USA	BEI Resources, NR-55611, Lot: 70045238	p1	1.1 × 10^7^
hCoV-19/USA/HI-CDC-4359259-001/2021	omicron	B.1.1.529	HI, USA	BEI Resources, NR-56475 Lot: 70049691	p1	2.1 × 10^7^
**hCoV-19/USA/MD-HP40900/2022**	omicron-XBB.1.5	B.1.1.529-XBB.1.5	MD, USA	BEI Resources, NR-59104 Lot: 70057837	p1	7.2 × 10^7^
**MA10 (mouse adapted USA-WA1/2020 backbone)**	B.1 MA-10	WA1	N/A	BEI Resources, NR-55329 Lot: 70043185	p1	2.3 × 10^7^

**Table 2:** Primary antibody list and conditions. Fluorophores (fluor) used: AF488; AF594; and AF694; or Biotinylated (B) antibody.

Antigen Recognized	Host Species and Format	Source and Catalog Number	RRID	Protocol
CD3	Rat monoclonal	BioLegend (100202)	AB_312659	(1:400) with fluor-conjugated 2nd antibody
CD68	Rat monoclonal	Invitrogen (14-0681-82)	AB_2572857	(1:100) with fluor-conjugated 2nd antibody
Iba1	Goat polyclonal	Novus Biologicals (NB100-1028)	AB_521594	(1:200) with B DαGt with fluor-SA
Ly6g	Rat monoclonal	Invitrogen (16-9668-82)	AB_2573128	(1:200) with fluor-conjugated 2nd antibody
SARS-CoV-2 nucleocapsid protein (NP)	Rabbit monoclonal	Sino Biological (40143-R019)	AB_2827973	(1:5000) with fluor-conjugated 2nd antibody
NeuN	Mouse monoclonal	Protein Tech (66836-1-Ig)	AB_2882179	(1:500) with B DαM with fluor-SA
GFAP (Ga5)	Mouse monoclonal	Cell Signaling Technology (3670)	AB_561049	(1:500) with fluor-conjugated 2nd antibody

## Data Availability

Raw data are all available as Supplementary Materials. Raw images are available at the following links: 10.5281/zenodo.14706868 (Figure 2, Figure 3, Figure 5, Figure 6, Figure 7, Figure 8 and Figure 9) and 10.5281/zenodo.14954785 (Figure 4, Figure 10, Figure 11 and Figure 12).

## Supplementary Materials

The following supporting information can be downloaded at https://www.mdpi.com/article/10.3390/v17040500/s1. Table S1: Statistical comparisons in mean body weights by day post-inoculation in K18 mice inoculated with different SARS-CoV-2 variants analyzed by 2-way ANOVA. ns is not significant, *p* > 0.05, * is *p* < 0.05, ** is *p* < 0.01, *** is *p* < 0.001, **** is *p* < 0.0001; Table S2. Statistical comparisons in mean SARS-CoV-2 titers by day post-inoculation in K18 mice inoculated with different SARS-CoV-2 variants analyzed by 2-way ANOVA. ns is not significant, *p* > 0.05, * is *p* < 0.05, ** is *p* < 0.01, *** is *p* < 0.001, **** is *p* < 0.0001; Table S3. Statistical comparisons of mean SARS-CoV-2 titers in swab and tissues in K18 mice inoculated with different SARS-CoV-2 variants analyzed by 2-way ANOVA. ns is not significant, *p* > 0.05, * is *p* < 0.05, ** is *p* < 0.01, *** is *p* < 0.001, **** is *p* < 0.0001; Table S4. Statistical comparisons in mean SARS-CoV-2 titers over time in C57BL/6J mice inoculated with different SARS-CoV-2 variants analyzed by 2-way ANOVA. ns is not significant, *p* > 0.05, * is *p* < 0.05, ** is *p* < 0.01, *** is *p* < 0.001, **** is *p* < 0.0001; Table S5. Statistical comparisons of mean SARS-CoV-2 titers in swabs and tissues in C57BL/6J mice inoculated with different SARS-CoV-2 variants analyzed by 2-way ANOVA. ns is not significant, *p* > 0.05, * is *p* < 0.05, ** is *p* < 0.01, *** is *p* < 0.001, **** is *p* < 0.0001.
