# Supplementary material for: Severe Acute Respiratory Syndrome Coronavirus 2 Variant Infection Dynamics and Pathogenesis in Transgenic K18-hACE2 and Inbred Immunocompetent C57BL/6J Mice
Source: Viruses. 2025 Mar 30;17(4):500. doi: 10.3390/v17040500 (PMC12031173; doi:10.3390/v17040500)
Supplement: Supplementary file 1 [file viruses-17-00500-s001.zip › viruses-3463320-supplementary.pdf]

SUPPLEMENTARY MATERIALS

Supplemental Table S1: Statistical comparisons in mean body weights by day post-inoculation in K18 mice inoculated with different SARS-CoV-2 variants analyzed by 2-way ANOVA. ns is not significant, p>0.05, * is p<0.05, ** is p<0.01, *** is p<0.001, **** is p<0.0001.

	**Day post inoculation**						
**Variant Comparison**	**1**	**2**	**3**	**4**	**5**	**6**	**Group losing more weight** (Superscript indicates day)
**DPBS vs B.1**	ns	ns	ns	****	****	****	B.1
**DPBS vs alpha**	ns	***	****	****	****	****	alpha
**DPBS vs beta**	ns	****	****	****	****	****	beta
**DPBS vs delta**	ns	ns	ns	***	****	****	delta
**DPBS vs omicron**	ns	***	****	****	*	ns	omicron
**B.1 vs alpha**	ns	ns	****	****	ns	ns	alpha
**B.1 vs beta**	ns	**	****	*	ns	ns	beta
**B.1 vs delta**	ns	ns	ns	ns	ns	ns	n/a
**B.1 vs omicron**	ns	*	****	ns	**	****	omicron[3,4,] B.1 [5,6]
**B.1 vs omicron-XBB.1.5**	ns	****	****	****	ns	***	omicron-XBB.1.[2,3,4], B.1[6]
**alpha vs beta**	ns	ns	ns	**	***	ns	alpha
**alpha vs delta**	ns	ns	****	****	**	ns	alpha
**alpha vs omicron**	ns	ns	ns	**	****	****	alpha
**alpha vs omicron-XBB.1.5**	ns	**	ns	ns	***	***	omicron-XBB.1.5[2], alpha[5,6]
**beta vs delta**	ns	**	****	**	ns	ns	beta
**beta vs omicron**	ns	ns	ns	ns	ns	**	beta
**beta vs omicron-XBB.1.5**	ns	ns	ns	ns	ns	ns	n/a
**delta vs omicron**	ns	*	***	ns	ns	****	omicron[2,3,] delta[6]
**delta vs omicron-XBB.1.5**	ns	****	****	****	ns	*	omicron-XBB.1.5[2,3,4], delta[6]
**omicron vs omicron-XBB.1.5**	ns	ns	ns	*	ns	ns	omicron-XBB.1.5

Supplemental Table S2. Statistical comparisons in mean SARS-CoV-2 titers by day post-inoculation in K18 mice inoculated with different SARS-CoV-2 variants analyzed by 2-way ANOVA. ns is not significant, p>0.05, * is p<0.05, ** is p<0.01, *** is p<0.001, **** is p<0.0001.

	**Oropharyngeal swab**	**Lung**			**Brain**
	**Day post inoculation**				
**Strain**	**1 vs 2**	**2 vs 4**	**2 vs 6**	**4 vs 6**	**4 vs 6**
**B.1**	ns	ns	ns	ns	***
**alpha**	****	ns	**	ns	**
**beta**	ns	ns	***	ns	*
**delta**	****	*	**	ns	ns
**omicron**	ns	ns	ns	ns	ns
**omicron-XBB.1.5**	****	ns	ns	ns	ns

Supplemental Table S3. Statistical comparisons of mean SARS-CoV-2 titers in swab and tissues in K18 mice inoculated with different SARS-CoV-2 variants analyzed by 2-way ANOVA. ns is not significant, p>0.05, * is p<0.05, ** is p<0.01, *** is p<0.001, **** is p<0.0001.

Tissue	Day post-inoculation	Strain	B.1	alpha	beta	delta	omicron
		alpha	ns				
		beta	ns	ns			
	1	delta	*	ns	ns		
		omicron	****	****	****	****	
Oropharyngeal swab		omicron-XBB.1.5	****	****	****	****	ns
		alpha	****				
		beta	ns	****			
	2	delta	****	**	****		
		omicron	****	**	****	ns	
		omicron-XBB.1.5	ns	****	ns	****	****
		alpha	ns				
		beta	ns	ns			
	2	delta	ns	ns	ns		
		omicron	*	ns	ns	ns	
		omicron-XBB.1.5	ns	*	ns	ns	***
Lung		alpha	ns				
		beta	ns	ns			
	4	delta	ns	ns	ns		
		omicron	ns	ns	ns	ns	
		omicron-XBB.1.5	ns	*	ns	*	ns
		alpha	ns				
		beta	ns	ns			
	6	delta	ns	ns	ns		
		omicron	ns	ns	ns	ns	
		omicron-XBB.1.5	ns	**	**	**	ns
		alpha	**				
		beta	ns	**			
	4	delta	ns	ns	ns		
		omicron	ns	****	ns	**	
Brain		omicron-XBB.1.5	ns	****	ns	**	ns
		alpha	ns				
		beta	ns	ns			
	6	delta	ns	ns	ns		
		omicron	****	****	**	****	
		omicron-XBB.1.5	**	****	ns	**	ns

Supplemental Table S4. Statistical comparisons in mean SARS-CoV-2 titers over time in C57BL/6J mice inoculated with different SARS-CoV-2 variants analyzed by 2-way ANOVA. ns is not significant, $p>0.05$, * is $p<0.05$, ** is $p<0.01$, *** is $p<0.001$, **** is $p<0.0001$.

	**Oropharyngeal swab**			**Trachea**				**Lung**	
**Days compared**	**1 vs 2**		**2 vs 4**	**2 vs 6**	**4 vs 6**		**2 vs 4**	**2 vs 6**	**4 vs 6**
**alpha**	ns		ns	ns	ns		ns	ns	ns
**beta**	ns		****	****	ns		****	****	ns
**omicron-XBB.1.5**	ns		ns	****	**		ns	ns	ns
**B.1 MA-10**	ns		ns	ns	ns		*	****	**

Supplemental Table S5. Statistical comparisons of mean SARS-CoV-2 titers in swabs and tissues in C57BL/6J mice inoculated with different SARS-CoV-2 variants analyzed by 2-way ANOVA. ns is not significant, p>0.05, * is p<0.05, ** is p<0.01, *** is p<0.001, **** is p<0.0001.

Tissue	Day post-inoculation	Variant	alpha	beta	omicron-XBB.1.5
		beta	****		
swab	1	omicron-XBB.1.5	*	****	
		B.1 MA-10	ns	****	*
		beta	****		
swab	2	omicron-XBB.1.5	ns	****	
		B.1 MA-10	ns	****	ns
		beta	****		
trachea	2	omicron-XBB.1.5	**	ns	
		B.1 MA-10	ns	****	**
		beta	ns		
trachea	4	omicron-XBB.1.5	*	ns	
		B.1 MA-10	ns	ns	*
		beta	ns		
trachea	6	omicron-XBB.1.5	ns	ns	
		B.1 MA-10	ns	ns	ns
		beta	**		
lung	2	omicron-XBB.1.5	ns	***	
		B.1 MA-10	**	ns	***
		beta	ns		
lung	4	omicron-XBB.1.5	ns	ns	
		B.1 MA-10	*	ns	ns
		beta	ns		
lung	6	omicron-XBB.1.5	ns	ns	
		B.1 MA-10	ns	ns	ns
